# A new digital method of data collection for spatial point pattern analysis in grassland communities

**DOI:** 10.1002/ece3.6512

**Published:** 2020-06-28

**Authors:** Xinting Wang, Chao Jiang, Chengzhen Jia, Yang Tai, Yali Hou, Weihua Zhang

**Affiliations:** ^1^ School of Energy and Power Engineering Inner Mongolia University of Technology Hohhot China; ^2^ Key of Laboratory of Grassland Ecology and Restoration Institute of Grassland Research Ministry of Agriculture Chinese Academy of Agriculture Sciences Hohhot China; ^3^ Ecological and Agricultural Meteorological Center of Inner Mongolia Hohhot China; ^4^ Inner Mongolia Coral Environmental Technology Co., Ltd Hohhot China

**Keywords:** digital photographs, geographical information system, point pattern, sampling technique, stress gradient hypothesis

## Abstract

A major objective of plant ecology research is to determine the underlying processes responsible for the observed spatial distribution patterns of plant species. Plants can be approximated as points in space for this purpose, and thus, spatial point pattern analysis has become increasingly popular in ecological research. The basic piece of data for point pattern analysis is a point location of an ecological object in some study region. Therefore, point pattern analysis can only be performed if data can be collected. However, due to the lack of a convenient sampling method, a few previous studies have used point pattern analysis to examine the spatial patterns of grassland species. This is unfortunate because being able to explore point patterns in grassland systems has widespread implications for population dynamics, community‐level patterns, and ecological processes. In this study, we developed a new method to measure individual coordinates of species in grassland communities. This method records plant growing positions via digital picture samples that have been sub‐blocked within a geographical information system (GIS). Here, we tested out the new method by measuring the individual coordinates of *Stipa grandis* in grazed and ungrazed *S. grandis* communities in a temperate steppe ecosystem in China. Furthermore, we analyzed the pattern of *S. grandis* by using the pair correlation function *g*(*r*) with both a homogeneous Poisson process and a heterogeneous Poisson process. Our results showed that individuals of *S. grandis* were overdispersed according to the homogeneous Poisson process at 0–0.16 m in the ungrazed community, while they were clustered at 0.19 m according to the homogeneous and heterogeneous Poisson processes in the grazed community. These results suggest that competitive interactions dominated the ungrazed community, while facilitative interactions dominated the grazed community. In sum, we successfully executed a new sampling method, using digital photography and a geographical information system, to collect experimental data on the spatial point patterns for the populations in this grassland community.

## Graphical abstract

A major objective of plant ecology research is to determine the underlying processes responsible for the observed spatial distribution patterns of plant species. Plants can be approximated as points in space for this purpose, and thus, spatial point pattern analysis has become increasingly popular in ecological research. We successfully executed a new sampling method, using digital photography and a geographical information system, to collect experimental data on the spatial point patterns for the populations in this grassland community.

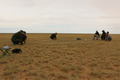

## 1 INTRODUCTION

The nonrandom spatial distribution of a species can have a certain amount of predictability (Dale & Zbigniewicz, 1995). Since the initial work of Greig‐Smith (1952, 1961), interest in studying the population spatial patterns in natural communities has increased (Condit, 2000; Dale & MacIsaac, 1989; Galiano, 1983; Greig‐Smith, 1987; Jácome‐Flores, Delibes, Wiegand, & Fedriani, 2016; Kershaw, 1963; Pielou, 1968; Ver Hoef, Cressie, & Glenn‐Lewin, 1993; Wang et al., 2010; Wiegand, Gunatilleke, & Gunatilleke, 2007). Individual spatial patterns within populations can offer insights for past and present processes, and these recognized patterns may also influence future processes (Law et al., 2009). In short, spatial patterns often reveal ecological processes (Greig‐Smith, 1979; Leps, 1990; Watt, 1947; Wiegand Gunatilleke, & Gunatilleke, 2007; Wiegand, Gunatilleke, Gunatilleke, & Okuda, 2007), but it should be noted that different ecological processes may lead to the same spatial pattern. For instance, the Neyman–Scott process, also called the Poisson cluster process, can effectively model both the dissemination of offspring around parents and the species response to habitat heterogeneity. Hence, ecological processes cannot be inferred from spatial patterns without additional external information. Thus, the underlying processes responsible for the observed spatial patterns are of key interest to ecologists (Liehold & Gurevitch, 2002; McIntire & Fajardo, 2009; Tilman & Kareiva, 1997; Tuda, 2007).

Recently, there has been rapid development in spatial pattern methodologies (Dale, 1999; Diggle, 2003, 2013; Ripley, 1981; Stoyan & Stoyan, 1994). Plants can be approximated as points in space, and thus, spatial point pattern analysis has become increasingly popular in ecological research (Velázquez, Martínez, Getzin, Moloney, & Wiegand, 2016). Now, the distribution of point pair distances is being used to describe the characteristics of point patterns with second‐order statistics (Diggle, 2013; Velázquez et al., 2016; Wiegand & Moloney, 2014), such as the pair correlation function or Ripley's *K* (Ripley, 1981), which can describe a range of distances and also detect mixed patterns.

The basic data unit for point pattern analysis is a point location of an ecological object in some study region. All that is necessary is determining its coordinate within the observation window, in this case, a 2D *x* and *y* location. In short, the key to performing point pattern analysis is the ability to collect these data. Various approaches can be applied to obtain the coordinates of ecological objects in a given study region. For example, a grid may be used in the field, and the position of an ecological object can be determined by measuring its distance and direction from the nearest node of the grid (Wiegand & Moloney, 2014). The location of objects can also be determined by using survey equipment or GPS devices (Chacón‐Labella, de la Cruz, & Escudero, 2016). A third approach is to determine the locations of objects indirectly from aerial photographs or satellite images, potentially allowing a very broad area to be sampled (e.g., Gil, Lobo, Abadi, Silva, & Calado, 2013; Moustakas et al., 2006; Nelson, Niemann, & Wulder, 2002). In all instances of point pattern analysis, it is critical to sample as complete an area as possible. Using these sampling methods, ecologists have often sought to comprehend the spatial distribution of a population by examining forests or shrubs (Velázquez et al., 2016) due to the great availability of data in these ecosystems (Law et al., 2009).

Although many studies have examined spatial patterns in grasslands (Greig‐Smith, 1987; Kershaw & Looney, 1985; Krahulec, Agnew, Agnew, & Willems, 1990; Purves & Law, 2002), only a few have used point data to analyze plant distribution (Brix & Chadoeuf, 2002). This paucity might be due to the lack of a convenient sampling method by which to collect these data in grassland communities. Some older methods require a lot of labor, and others are no longer applicable. However, understanding point patterns in grassland systems has widespread implications for population dynamics, community‐level patterns, and ecological processes. Therefore, a convenient method for determining point locations accurately is necessary in grassland communities. Recent advances in digital photography and image analysis of software provide new opportunities for improved spatial data collection and analysis. Digital cameras provide high‐resolution pictures that can be quickly transferred to a computer for analysis. Geographical information system (GIS) and image analysis software and technology make it possible to objectively quantify spatial information from digital images in a repeatable and timely manner. Thus, we opted to design a new sampling method here to make possible the measurement of individual locations of species in grassland communities and the subsequent point pattern analysis. This new method involves digital photographs and a geographical information system (GIS), and we expect that it will help ecologists study the underlying processes that create the observable spatial patterns in grassland communities.

## 2 SAMPLING METHODS

### 2.1 Data collection using digital photographs and GIS

**FIGURE 1:**
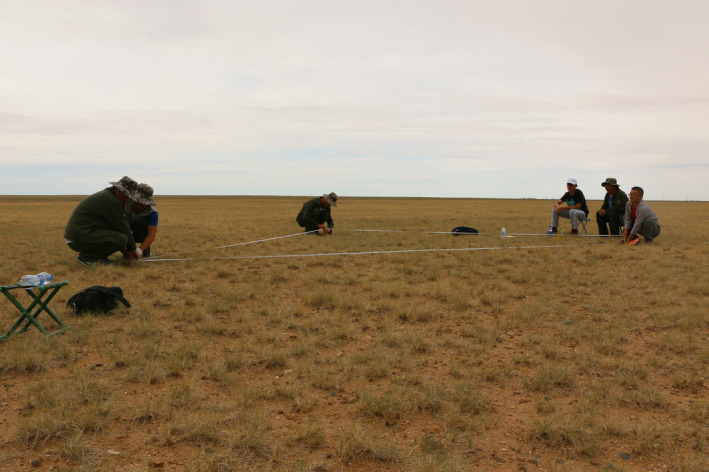
A flat 5 m × 5 m sampling block was chosen in an objective community in the desert steppe

**FIGURE 2:**
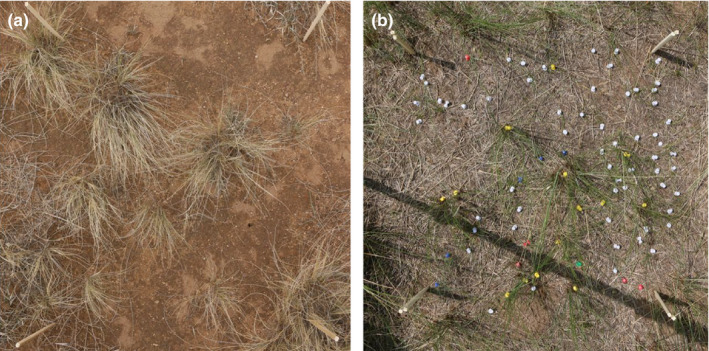
Digital pictures of 50 cm × 50 cm sub‐blocks in the steppe communities, marked by bamboo chopsticks. (a) In the desert steppe. (b) Individuals uniquely marked and distinguished from different populations by using pushpins of different colors in the typical steppe

A flat 5 m × 5 m sampling block was chosen in a study grassland community and divided with bamboo chopsticks into 100 sub‐blocks of 50 cm × 50 cm (Figure 1). A digital camera was then mounted to a telescoping stake and positioned in the center of each sub‐block to photograph vegetation within a 0.25‐m^2^ area. Pictures were taken 1.75 m above the ground at an approximate downward angle of 90° (Figure 2). Automatic camera settings were used for focus, lighting, and shutter speed. After photographing the plot as a whole, photographs were taken of each individual plant in each sub‐block. In order to identify each individual plant from the digital images, each plant was uniquely marked before the pictures were taken (Figure 2b).

**FIGURE 3:**
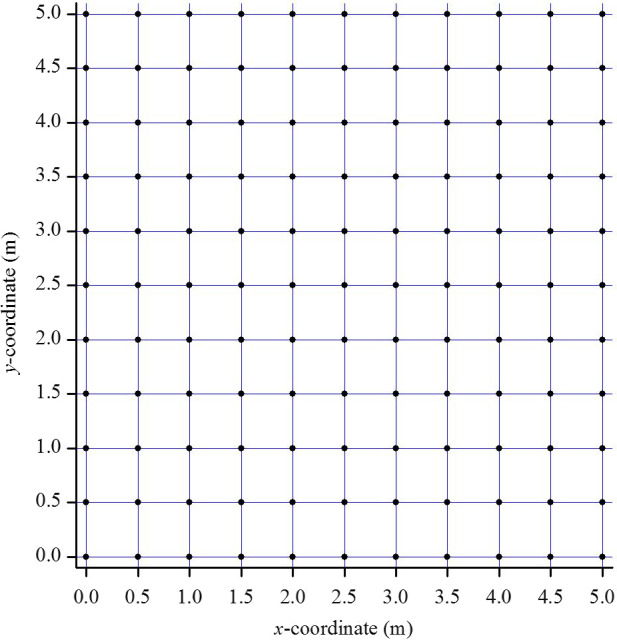
Coordinates of the control points in a 5 m × 5 m study plot. (●) control points (the vertexes of each sub‐block)

Digital images were imported into a computer as JPEG files, and the position of each plant in the pictures was determined using GIS. This involved four steps: (1) A reference frame (Figure 3) was established using R2V software to designate control points, or the four vertexes of each sub‐block (Figure S1), so that all plants in each sub‐block were within the same reference frame. The parallax and optical distortion in the raster images was then geometrically corrected based on these selected control points; (2) maps, or layers in GIS terminology, were set up for each species as PROJECT files (Figure S2), and all individuals in each sub‐block were digitized using R2V software (Figure S3). For accuracy, the digitization of plant individual locations was performed manually; (3) each plant species layer was exported from a PROJECT file to a SHAPE file in R2V software (Figure S4); (4) Finally, each species layer was opened in ArcGIS software in the SHAPE file format, and attribute data from each species layer were exported into ArcGIS to obtain the precise coordinates for each species. This last phase involved four steps of its own, from adding the data (Figure S5), to opening the attribute table (Figure S6), to adding new *x* and *y* coordinate fields (Figure S7), and to obtaining the *x* and *y* coordinates and filling in the new fields (Figure S8).

### 2.2 Data reliability assessment

**FIGURE 4:**
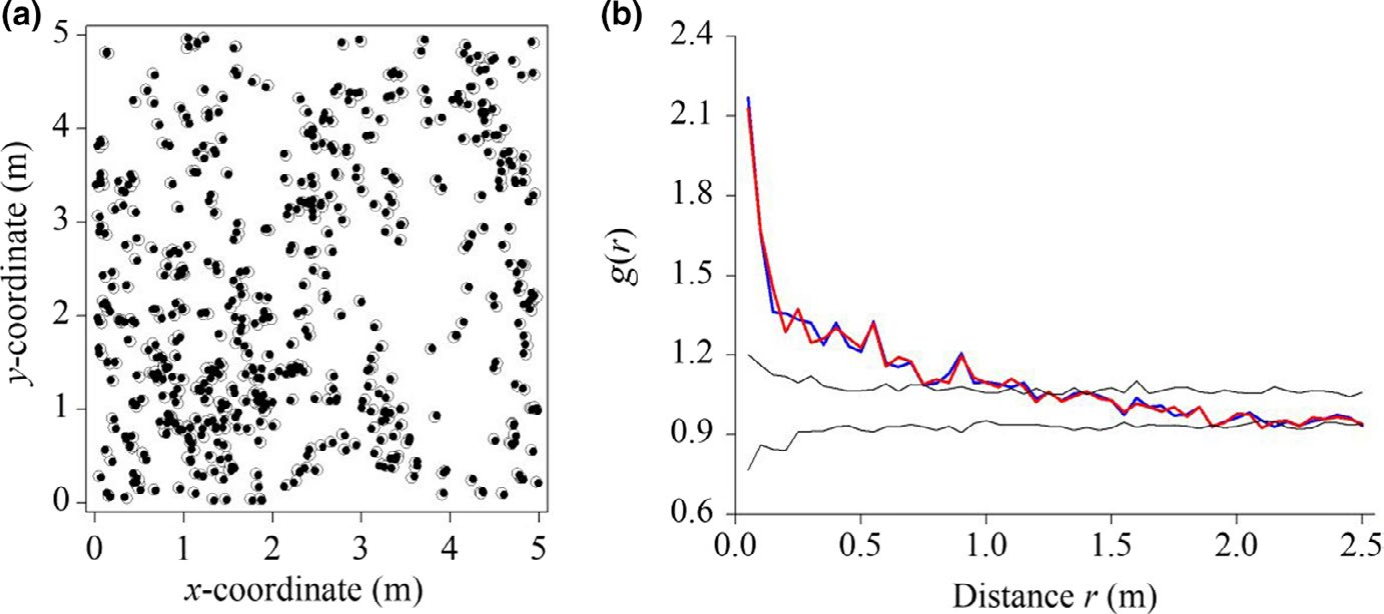
Example data collected to assess the reliability of the method developed in this study. (a) Mapped point pattern of observed data, as measured with a ruler (○) and by using digital photographs and geographical information system (●). (b) Analysis of the spatial pattern of observed data in the 5 m × 5 m study plot collected with a ruler (red lines) and with our new method (digital photographs and geographical information system) (blue lines). The black lines show the confidence limits of the pair correlation functions. The confidence limits were constructed using the highest and lowest *g*(*r*) from 199 replicates of the homogeneous Poisson process (complete spatial randomness)

To determine the accuracy of our new method, we measured the individual locations of *Leymus chinensis*, a perennial rhizome grass, in representative community blocks 5 m × 5 m in size in typical steppe habitat in the Inner Mongolia Autonomous Region of China in July 2010 (Figure 4a). As our standard for comparison, we used a ruler to measure the individual coordinates of *L. chinensis*. We tested for significant differences between (1) the coordinates of *L. chinensis*, as measured with our new method and with the ruler, and (2) the pair correlation function *g* of *L. chinensis*, as measured with our new method and with the ruler (see Section 3.2). If (1) the coordinates of *L. chinensis*, as measured with our new method and with the ruler, and (2) the pair correlation function *g* of *L. chinensis*, as measured with our new method and with the ruler, did not differ significantly, then we could conclude that our new method of measuring the coordinates of *L. chinensis* was reliable.

We compared the results using a *t* test (Table 1). We found no significant differences in either (1) the coordinates of *L. chinensis* or (2) the pair correlation function *g* of *L. chinensis*. Further, we compared the pattern characteristics of *L. chinensis* when measured by our new method against the ruler measurements using a null model. We found that the two pattern characteristics of *L. chinensis* did not differ significantly based on the homogeneous Poisson process or complete spatial randomness (Figure 4b). Thus, we concluded that the data obtained using our new method were reliable enough to perform point pattern analysis with a null model in grassland communities.

**TABLE 1:** Comparison of spatial pattern results measured by different methods: the standard ruler and the method developed in this study (digital photographs and geographical information system) (*p* ＜ .05). This comparison was conducted to assess the accuracy of our new method

	Mean	Std. deviation	Std. error mean	*t*	*df*	*p*
*x*_1_–*x*_2_	0.0019648	0.0287637	0.0012069	1.628	567	.104
*y*_1_–*y*_2_	−4.2957746E−4	0.0280268	0.0011760	−0.365	567	.715
*g*(*r*)_1_–*g*(*r*)_2_	−1.384000000E−4	0.009593630	0.001356744	−0.102	49	.919

Note

*x*, abscissa about point; *y*, ordinate about point; *g*(*r*), the pair correlation function; subscript 1, measured by ruler; subscript 2, measured by the new method developed in this study (digital photographs and geographical information system).

## 3 EXAMPLE

### 3.1 Study sites

The study site is a permanent field site within the Inner Mongolia Grassland Ecosystem Research Station (116°42′E, 43°38′N), located in the Xilin River Basin in the Inner Mongolia Autonomous Region of China. The 500 m × 500 m plot is dominated by perennial bunchgrass *Stipa grandis* and has been fenced‐off since 1979 to prevent grazing. At the time of exclosure, the site was considered to be in excellent condition, representative of an undisturbed climax steppe community (Bai, Han, Wu, Chen, & Li, 2004). The *Stipa grandis* community represents the most widely distributed type of grassland community across the Eurasia steppe region (Wang, Yong, & Liu, 1985). The area immediately outside of the site is open to large animal grazing and has become seriously degraded (Li, Wang, Liu, & Jiang, 2008; Wang, Liu, Hao, & Liang, 1996). In the region, dark chestnut soils are present, with a hummus layer 20–30 cm thick and a calcic layer at 50–60 cm below. Average annual temperature is 0°C with mean yearly precipitation around 350 mm. Interannual precipitation varies between 180 and 550 mm with 60%–80% falling during the summer season from June to August. This is typical of the temperate–semiarid climate of this region (Li et al., 2008). Annual potential evaporation ranges from 1,600 to 1,800 mm. Perennial plant species germinate following the rains that occur in early July, but the growing season technically runs from early April to late September.

**FIGURE 5:**
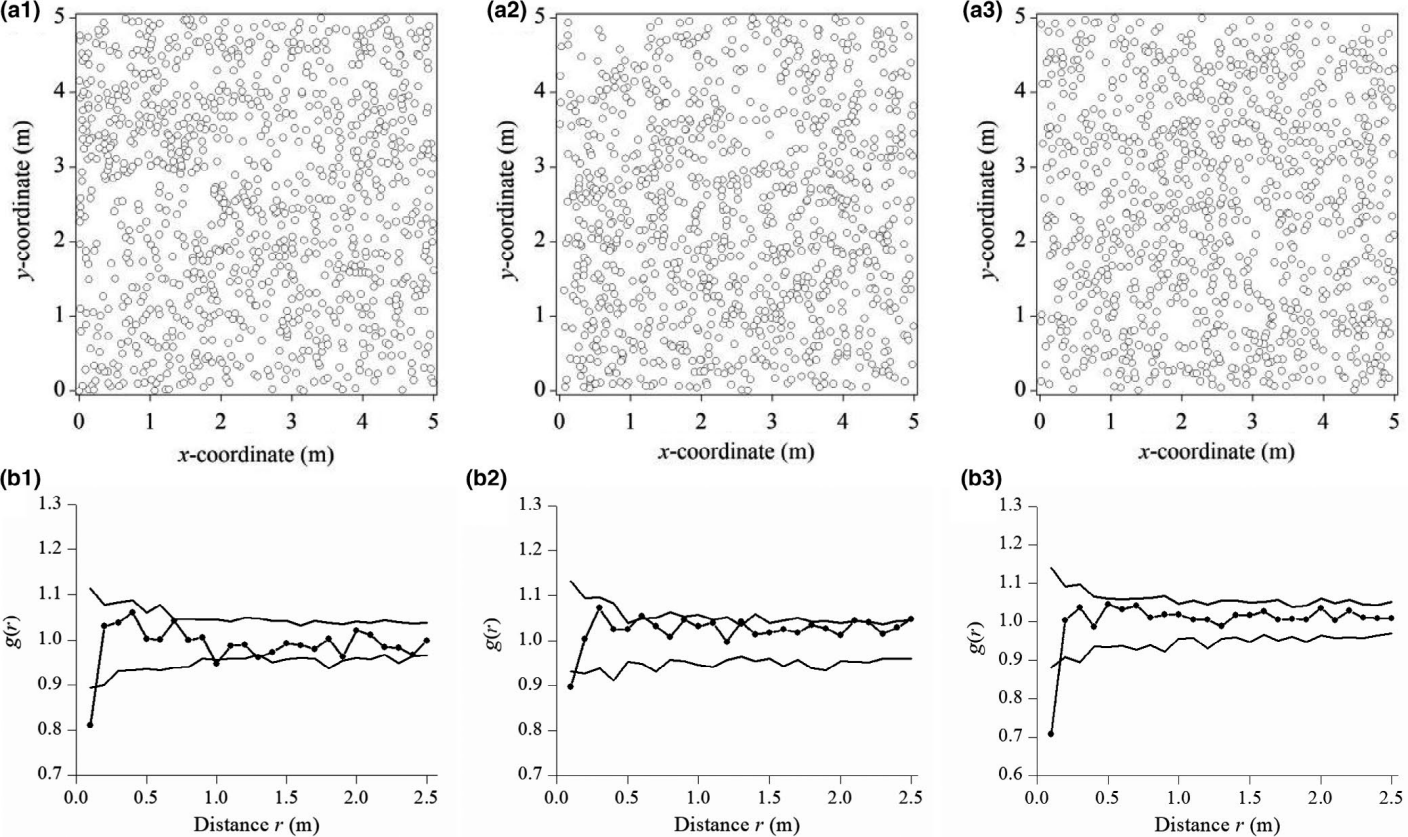
Spatial pattern analysis of *Stipa grandis* in the ungrazed *S. grandis* community in the typical steppe. (a) Point pattern; (b) the *g*(*r*) based on homogeneous Poisson (CSR). Confidence limits were constructed using the highest and lowest *g*(*r*) from 199 replicates of the null model (subscripts 1, 2, and 3 refer to plot replicates)

**FIGURE 6:**
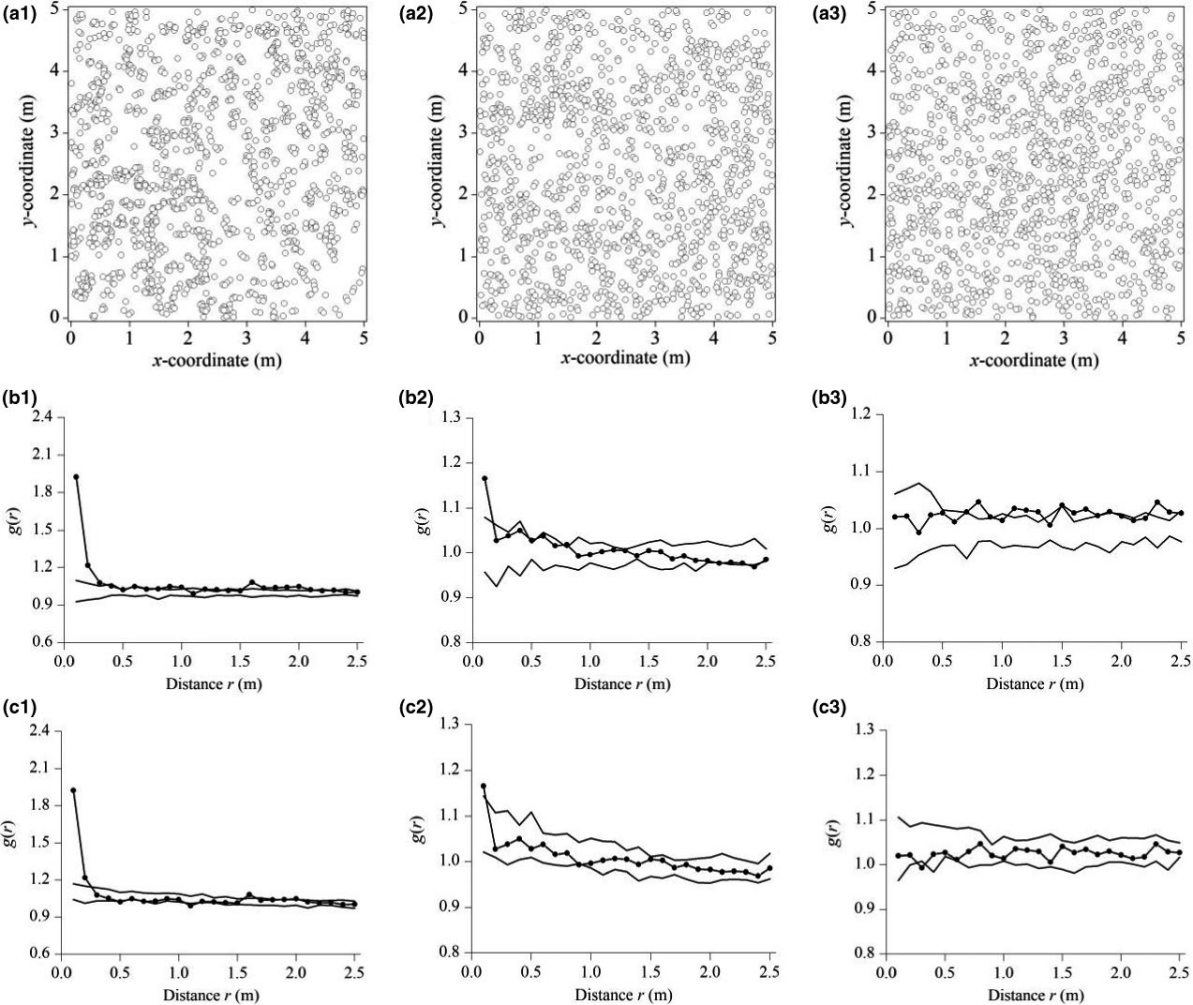
Spatial pattern analysis of *Stipa grandis* in the grazed *S. grandis* community in the typical steppe. (a) Point pattern; (b) the *g*(*r*) based on homogeneous Poisson (CSR); (c) the *g*(*r*) based on heterogeneous Poisson. Confidence limits were constructed using the highest and lowest *g*(*r*) from 199 replicates of the null model (subscripts 1, 2, and 3 refer to replicates)

In July 2017, we chose *S. grandis* as the study species and selected three 5 m × 5 m replicate community blocks within the site and three outside the site. In each of the replicate blocks, the individual locations of *S. grandis* were measured by using our new method with digital photographs and GIS (Figures 5a and 6a).

### 3.2 Data analysis

The pair correlation function *g*(*r*) (Stoyan & Penttinen, 2000; Stoyan & Stoyan, 1994) and Ripley's *K*(*r*) function (Ripley, 1976, 1977, 1981) are commonly used in the analysis of point patterns (Wiegand & Moloney, 2004). The pair correlation function *g*(*r*) and Ripley's *K*(*r*) function are both based on the distribution of distances between pairs of points. The *λK*(*r*) gives the expected number of points found within a distance *r* of an arbitrarily chosen point, where *λ* is the point process intensity of the pattern. Here, *K*(*r*) is based on all distances between points in the pattern. The pair correlation function *g*(*r*) is derived from the *K*(*r*) function, where *g*(*r*) = (2*πr*)^−1^d*K*(*r*)/d*r*. *K*(*r*) and *g*(*r*) are related to both the cumulative distribution function and the probability density function of distances between pairs of points (Diggle, 2003; Stoyan & Penttinen, 2000).

In this study, the pair correlation function *g*(*r*) was used to detect spatial point pattern characteristics. Unbiased interpretations of the pair correlation function *g*(*r*) require the selection of an appropriate null model that addresses the specific biological questions being asked (Wiegand & Moloney, 2004). Here, we used the homogeneous Poisson process to detect regularity and aggregation in univariate patterns. For the homogeneous Poisson process, the intensity (*λ*) is homogeneous across a region. This ensures an equal probability of any point occurring at any location irrespective of the location of all the other points. Also, the number of points in an area W should follow a Poisson distribution with a mean of *λ*W. We also applied the heterogeneous Poisson process to determine the individual effects of habitat heterogeneity in the aggregation patterns. For the heterogeneous Poisson model, the density of points in the pattern is associated with certain environmental factors. Compared with the homogeneous Poisson process, the constant intensity is replaced by a function (*x*, *y*) that varies with location (*x*, *y*). Here, nonparametric methods are used to estimate the intensity function *λ*(x, y) directly from the data using smoothing techniques based on kernel estimators (Wiegand & Moloney, 2014). From observations made in the field prior to this study, we found that environmental conditions in this grazing community varied across scales of about 2.0 m (personal observation). Thus, we constructed the intensity function *λ*(*x*, *y*) using a bandwidth *R* = 1.0 m. This is particularly appropriate for situations in which no additional information on environmental variables is available because it allows for the detection of potential gradients in the intensity of points (Wiegand & Moloney, 2014).

All analyses were conducted using Programmatic version 2014 (Wiegand & Moloney, 2014). We used this program to compare our observed data with the two null models described above. Confidence limits were constructed using the highest and lowest *g*(*r*) from 199 replicates of the null model. This led to an approximate type I error rate of alpha = 0.01.

In our study, we analyzed the observed data for each of the three replicates and combined the three replicates in each site into a single weighted pair correlation function *g*(*r*) (Diggle, 2013; Wiegand & Moloney, 2014).

### 3.3 Results

**FIGURE 7:**
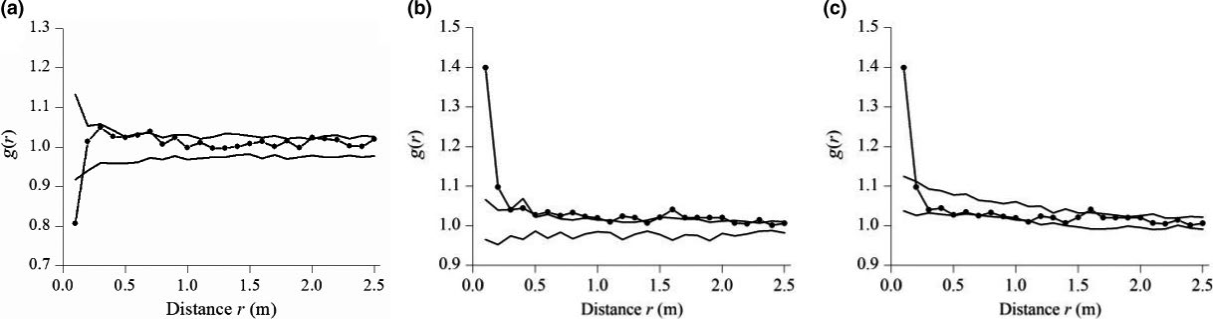
The weighted pair correlation function *g*(*r*) of *Stipa grandis* after combining the three replicates. (a) Based on homogeneous Poisson in the ungrazed *S. grandis* community; (b) based on homogeneous Poisson in the grazed *S. grandis* community; and (c) *g*(*r*) based on heterogeneous Poisson in the grazed *S. grandis* community

*Stipa grandis* individuals in plot replicate 1 of the ungrazed community were overdispersed according to the homogeneous Poisson process at 0–0.15 m and were random at 0.15–2.5 m (Figure 5b1). Similarly, individuals in plot replicate 2 were overdispersed at 0–0.13 m and were random at 0.13–2.5 m (Figure 5b2). Likewise, individuals in plot replicate 3 were overdispersed at 0–0.17 m and were random at 0.17–2.5 m (Figure 5b3). Overall, based on the weighted *g*(*r*) statistics by integrating the three different replicates in 5 × 5 m study plots, individuals of *S. grandis* were overdispersed at 0–0.16 m and were random at 0.16–2.5 m (Figure 7a).

In contrast, *Stipa grandis* individuals in plot replicate 1 of the grazed community were clustered according to the homogeneous Poisson process at 0–0.30 m and were random at 0.30–2.5 m (Figure 6b1). Similarly, individuals in plot replicate 2 were clustered at 0–0.17 m and were random at 0.17–2.5 m (Figure 6b2). However, individuals in plot replicate 3 were almost random at 0–2.5 m, except for some that were lightly clustered (Figure 6b3). Furthermore, based on the weighted *g*(*r*) statistics by integrating the three different replicates, individuals of *S. grandis* were aggregated at 0–0.30 m and were random at 0.30–2.5 m, according to the homogeneous Poisson process (Figure 7b).

For the grazed community, we opted to only use the heterogeneous Poisson process, which showed more aggregation than the homogeneous process. *Stipa grandis* individuals in plot replicate 1 were clustered according to the heterogeneous Poisson process at 0–0.25 m and fitted the heterogeneous Poisson process at 0.25–2.5 m (Figure 6c1). Similarly for individuals in plot replicate 2, they were clustered at 0–0.11 m and fitted the heterogeneous Poisson process at 0.11–2.5 m (Figure 6c2). In plot replicate 3 though, individuals of *S. grandis* fitted the heterogeneous Poisson process at 0–2.5 m (Figure 6c3). In sum, based on the weighted *g*(*r*) statistics by integrating the three different replicates, individuals of *S. grandis* were aggregated at 0–0.19 m according to the heterogeneous Poisson process and fitted the heterogeneous Poisson process at 0.19–2.5 m (Figure 7c).

## 4 DISCUSSION

### 4.1 Sampling method by using digital photographs and GIS

Recent improvements in digital photography and image analysis software have enabled better data collection and analysis of vegetation. For example, digital cameras capture high‐resolution pictures that can be analyzed on a computer. Luscier, Thompson, Wilson, Gorham, and Dragut (2006) used digital photographs and object‐based image analysis to collect vegetation data and to estimate vegetation ground cover percentage. Over large‐scale landscapes, remotely sensed datasets and GIS are conventionally used to classify land cover (Baatz et al., 2002). Digital images and GIS are also used to analyze spatial patterns based on point patterns, which are in some cases based on Ripley's *K* function (Malkinson, Kadmon, & Cohen, 2003). However, few past studies have considered spatial population patterns in grassland communities by using point pattern analysis on data collected by digital photography and GIS.

In this study, a new method has been proposed to measure spatial point patterns of grassland communities, including in the steppe of Inner Mongolian in China. This is a novel method in which the positions of each plant are accurately fixed in digital pictures of sub‐blocks using GIS.

With this new method, the spatial point patterns of plants in a grassland community can be more efficiently recorded than with a ruler, and the data have similar accuracy (Table 1, Figure 4). In fact, we used a ruler to evaluate the reliability of the data measured by the new method. The ruler method and the new method were performed simultaneously, and the data obtained by the ruler were considered the standard data. To ensure high accuracy with the ruler method, (1) we chose a flat 5 m × 5 m community block in the typical steppe in the Inner Mongolia Autonomous Region of China; (2) we chose *L. chinensis* as the target species and removed other species during measurement. *L. chinensis* is a rhizome grass, and its aboveground stem diameter is about 2 mm; (3) we carefully measured the individual coordinates of *L. chinensis* by using a steel ruler with a millimeter scale. We compared the results of the two methods using a *t* test (Table 1), and found no significant differences in either (1) the coordinates of *L. chinensis* or (2) the pattern of *L. chinensis* (Figure 4). Thus, the data obtained using our new method were reliable enough to perform point pattern analysis with a null model in grassland communities. At present, high‐precision GPS, such as Leica Viva GS15 (±1 cm), is the most convenient and accurate positioning method. However, when the distance between neighbors is less than 10 cm, the relative error with using high‐precision GPS is greater than 10%, which is not practical for measuring fine‐scale population patterns in grassland communities. Further, the high‐precision GPS cannot measure individual coordinates within 1 cm. Our new method digitizes points in a digital photograph (Figure S3) and does not have the above defects of high‐precision GPS.

Compared with other methods, our new method has its own advantages for spatial point pattern analysis in grassland communities. First of all, compared with traditional methods, such as the ruler, our method reduces fieldwork. Second, compared with remote sensing or unmanned aerial vehicle technologies that are mainly aimed at large‐scale population patterns in forests or shrubs, our new method is better able to detect small‐scale population patterns in grassland communities. Third, compared with high‐precision GPS, which is suitable for forests and shrubs, our new method is more accurate for grassland communities where plants are smaller and closer together. In addition, the current high‐precision GPS positioning system is expensive, which can make it economically infeasible for some studies. Finally, our new method captures digital photograph data, which may be revisited more easily than field samples and corrected a posteriori. Overall, our new method is fast and accurate at collecting data for population point analysis in grassland communities.

To ensure the highest accuracy when using our new method to determine population patterns, we offer a few recommendations.

This new method of combining digital photography with GIS is mainly suitable for herbaceous plant communities and communities of small shrubs, especially in grasslands. It has low feasibility for forests and large‐sized shrub communities.

With regard to digital cameras, the early Nikon D100 with 6.0 megapixels (lens focal length is 30 mm) was found to accurately measure the locations of plant individuals. As technology develops, newer digital cameras are expected to perform just as accurately. Researchers can choose suitable digital cameras according to their needs.

When determining the locations of individuals, the R2V software was chosen to establish control points and to digitize all individuals of each species in each sub‐block. The R2V software provides an easy and complete solution to digitize vector data from image sources such as digital photography, scanned maps and drawings, aerial photographs, and satellite imagery. Because of its flexibility and high accuracy, the software is well suited for GIS applications. In the ArcGIS software itself, establishing control points for our method can be challenging. Thus, we did not utilize the ArcGIS software to determine control points and to perform digitization. However, we did find that the measuring of individual coordinates could only be accomplished through the ArcGIS software.

We used 1.75 m as the shooting height in our experiment, but this height is not fixed. Researchers can adjust the height according to the needs of their experiment, as long as the picture is clear and the center of the viewfinder is aligned with the center of the subsample so that the four vertices of the subsample (i.e., four bamboo sticks) enter the viewfinder at the same time.

We selected a community block of 5 m × 5 m as the sampling size in our field experiment. However, the size of the sampling area may vary according to the research purpose and the species characteristics in the community. Other possible sizes may be 1 m × 1 m, 3 m × 3 m, 10 m × 10 m, or even 20 m × 20 m.

For the subquadrats in a sampling block, a size of 0.5 m x 0.5 m is reasonable. It can be useful for the subquadrats to divide the sampling area into some integer number of subareas, thereby making it easy to determine the coordinates of control points. Also important is that the larger the subquadrat, the higher the shooting height. Therefore, a subquadrat size such as 1 m × 1 m may be more difficult for a photographer to shoot.

When using this method to locate individuals in a population, it is necessary to accurately identify species from their digital photographs. In grassland communities especially, it can be difficult to distinguish species from each other in this way. Thus, it was important to mark each species uniquely before taking the pictures in order to facilitate their identification (Figure 2b). This can increase the amount of fieldwork. Moreover, when manually digitizing plant locations in GIS, caution must be taken to include every individual inside the subquadrat. To avoid digitizing plant individuals outside the subquadrat, a subquadrat boundary can be added through the vertex of the subquadrat using the R2V software (Figure S9).

### 4.2 Example point pattern analysis of *Stipa grandis* in a typical steppe

In the example, individuals of *S. grandis* were overdispersed in the ungrazed *S. grandis* community, according to the homogeneous Poisson process at small scales (at 0–0.16 m) (Figure 7a). This suggests that competitive interactions dominated the ungrazed *S. grandis* community (Grime, 1973). Meanwhile, in the grazed *S. grandis* community, individuals were clustered at 0–0.3 m (Figure 7b). This suggests that habitat heterogeneity can cause individual aggregation (Wiegand, Gunatilleke, & Gunatilleke, 2007). Therefore, we chose the heterogeneous Poisson process to investigate the individual effects of habitat heterogeneity in the grazed *S. grandis* community. We found that individual aggregation can be attributed to habitat heterogeneity in grazed communities at 0.19–0.3 m, while they are still clustered at 0–0.19 m (Figure 7c). Moreover, we also know that facilitation can bring about individual clustering (Jia, Dai, Shen, Zhang, & Wang, 2011). Facilitation is expected to be more intense than competition under high abiotic stress or consumer pressure (i.e., Bertness & Callaway, 1994; Callaway, 2007; Kikvidze, Suzuki, & Brooker, 2011). For example, the stress gradient hypothesis (SGH) predicts that facilitative interactions dominate under high‐stress conditions, and competitive interactions dominate in low‐stress conditions. In our study, grazed versus ungrazed communities can be viewed as stressful versus unstressful habitats because the region has been heavily disturbed by long‐term overgrazing by sheep (Wang et al., 1996). As a result, individual aggregation in grazed communities at 0–0.19 m could be a result of facilitation between individuals. Furthermore, the observed regularity versus aggregation of *S. grandis* at small scales in the primary and grazed communities, respectively, verified the stress gradient hypothesis (SGH). However, there are limitations in exploring the mechanisms of grazed versus ungrazed communities via point pattern analysis because spatial statistical techniques based on observational data cannot suggest causal relationships (Murrell, Purves, & Law, 2001). As such, further experimental work is necessary for manipulating these different processes.

In our example, three 5 m × 5 m community blocks were established for point pattern analysis. Although point pattern analysis via replicated sampling has been previously proposed (Diggle, 2013; Wiegand & Moloney, 2014), few studies have analyzed spatial population patterns in this way. Here, population patterns of *S. grandis* differed among the three replicated blocks in the grazed communities (Figure 6b,c). This illustrates that point pattern analysis of a single plot could be misleading for understanding the full population. It is more reliable to integrate the data of replicated plots into point pattern analysis with the pair correlation function *g*(*r*), which is a weighted average.

## 5 CONCLUSIONS

Our study successfully designed a new sampling method that employs digital photography and a geographical information system to collect experimental data for the spatial point patterns within a grassland community. Our example showed that the point pattern characteristics of populations in grassland communities can be revealed by our new sampling method and that the underlying processes that create the spatial patterns can be also explored by using null models.

## CONFLICT OF INTEREST

The authors declare no conflict of interest.

## AUTHOR CONTRIBUTION

**Xinting Wang:** Conceptualization (lead); Data curation (equal); Formal analysis (lead); Funding acquisition (supporting); Investigation (equal); Methodology (lead); Project administration (equal); Writing‐original draft (lead); Writing‐review & editing (equal). **Chao Jiang:** Data curation (equal); Funding acquisition (lead); Investigation (equal); Methodology (supporting); Project administration (equal); Writing‐original draft (supporting); Writing‐review & editing (equal). **Chengzhen Jia:** Data curation (equal); Investigation (equal). **Yang Tai:** Data curation (equal). **Yali Hou:** Data curation (equal). **Weihua Zhang:** Investigation (equal).

## Supporting information

Figure S1‐S11

## Data Availability

The data are available on Dryad: https://doi.org/10.5061/dryad.brv15dv70.
