# Supplementary material for: A new digital method of data collection for spatial point pattern analysis in grassland communities
Source: Ecol Evol. 2020 Jun 28;10(14):7851–60. doi: 10.1002/ece3.6512 (PMC7391328; doi:10.1002/ece3.6512)
Supplement: Supplementary file 1 — Figure S1‐S11 [file ECE3-10-7851-s001.doc]

**Supporting Information**


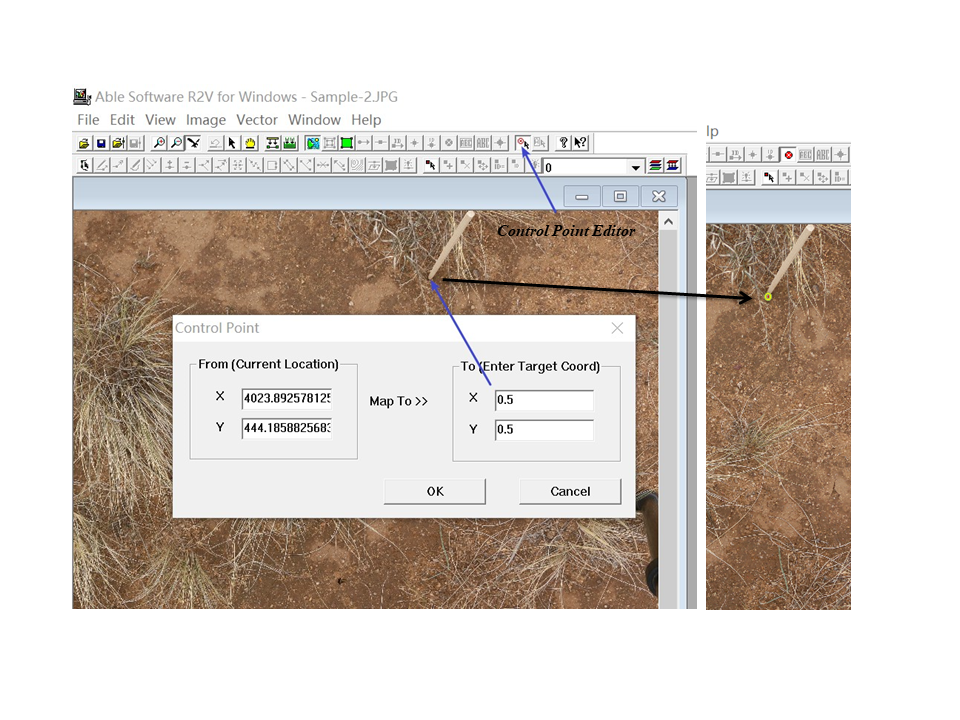


**Figure S1** Coordinates of the control points (the vertexes of each sub-block) were added to the digital images by using R2V 7.0 software.


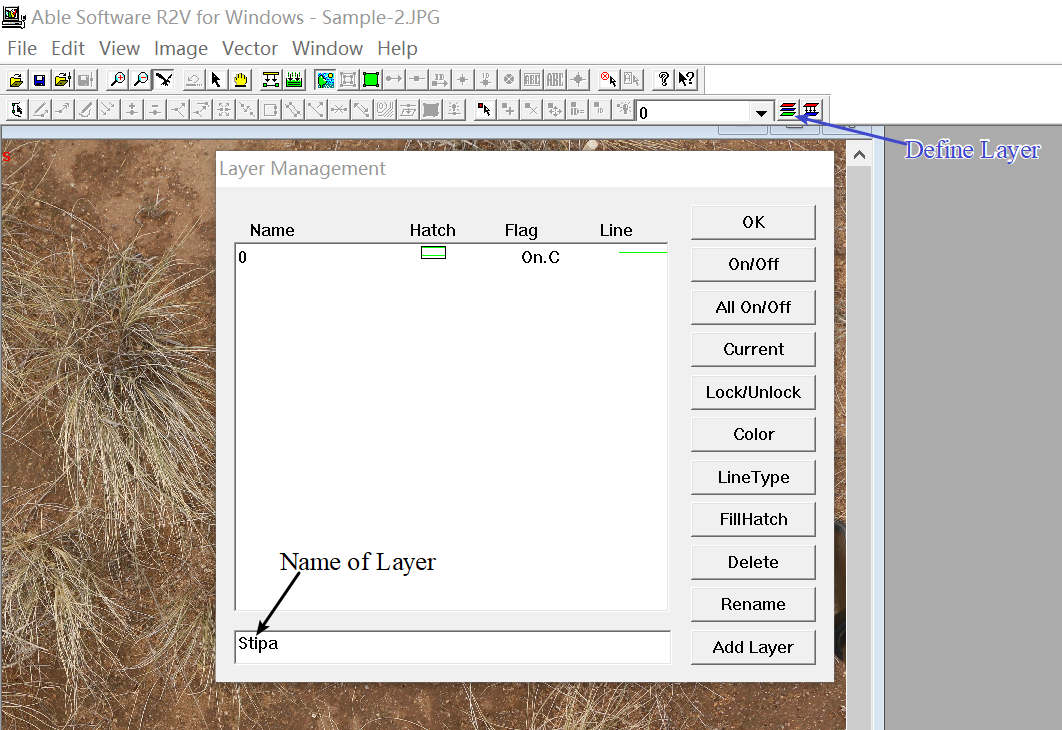


**Figure S2** Species layers were set up using R2V 7.0 software.


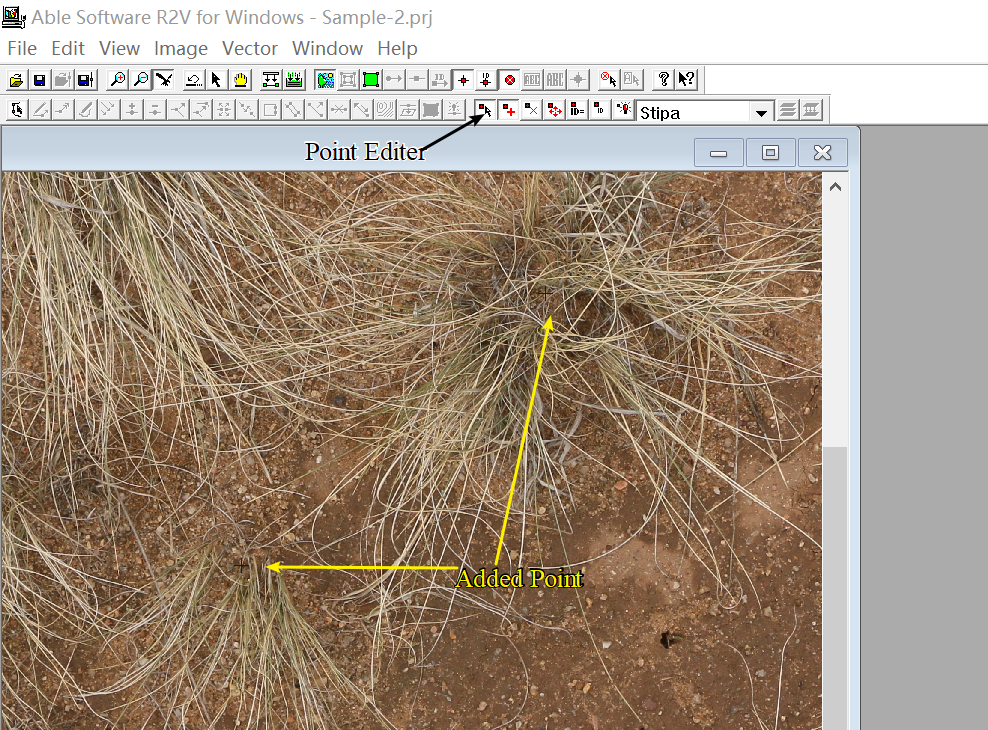


**Figure S3** The individuals of species in each sub-block were digitized using R2V 7.0 software.


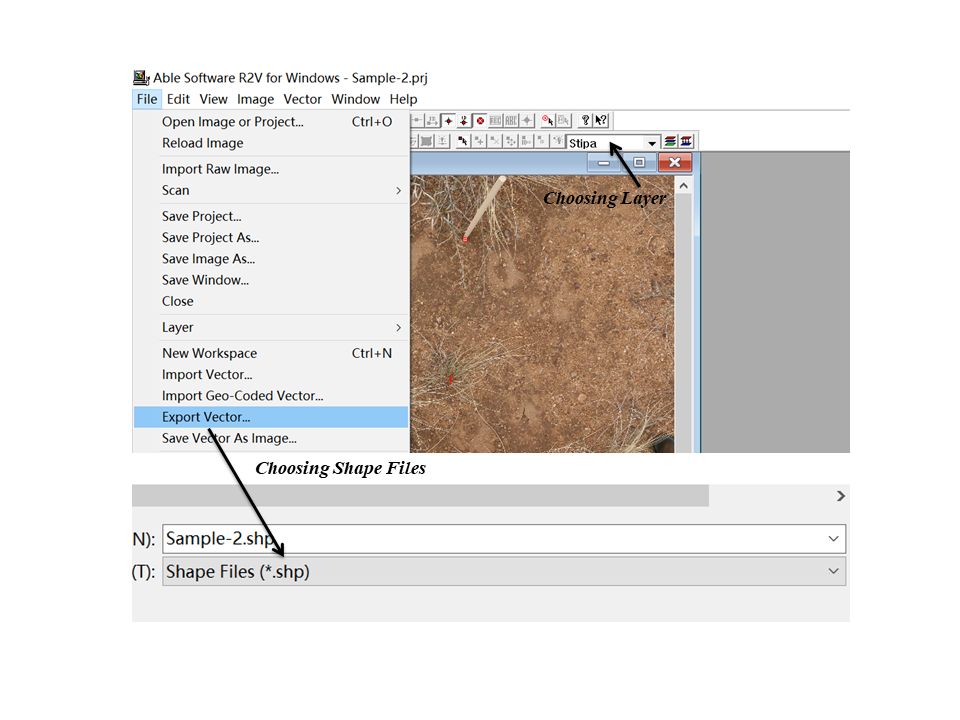


**Figure S4** The PROJECT file species layer was exported as a SHAPE file using R2V 7.0 software.


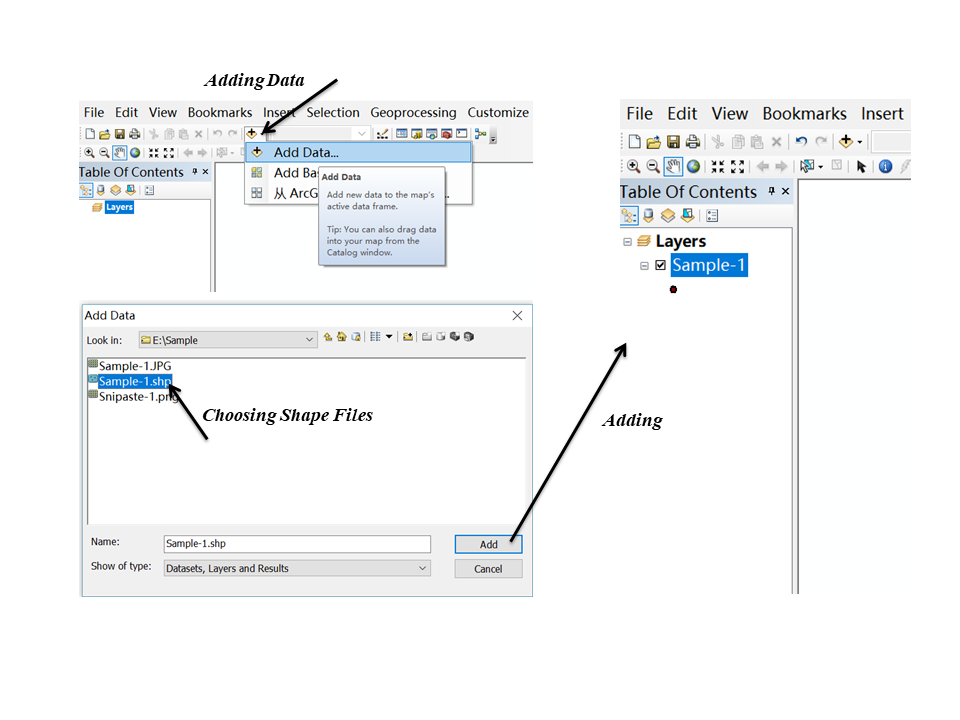


**Figure S5** The SHAPE file species layer was loaded into ArcMap using Arc GIS 10.3 software.


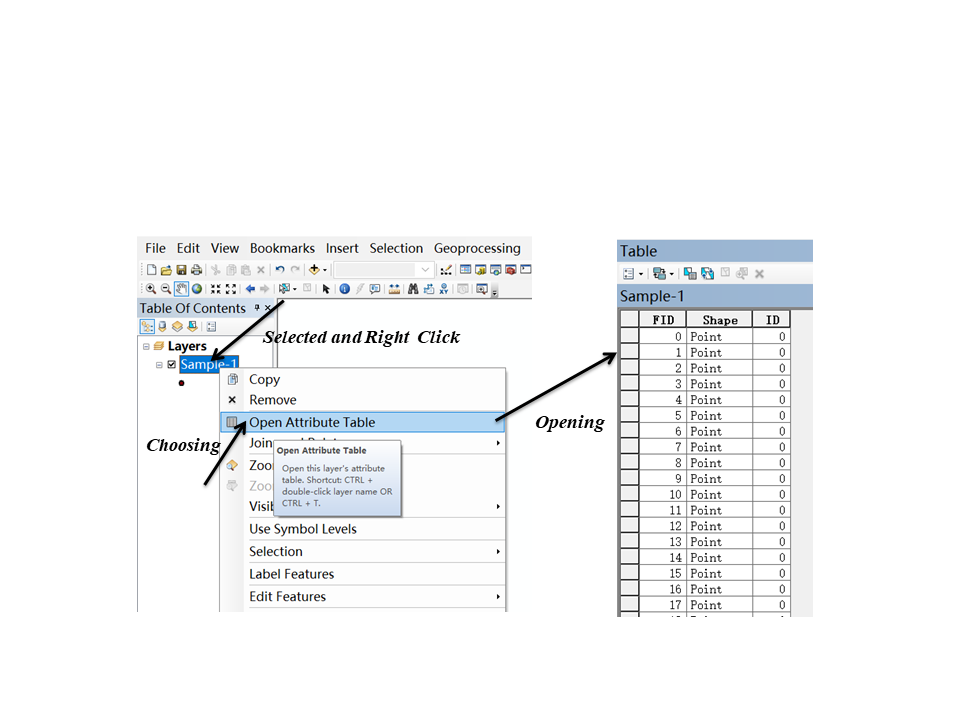


**Figure S6** The attribute table of the layer was opened in ArcMap using Arc GIS 10.3 software.


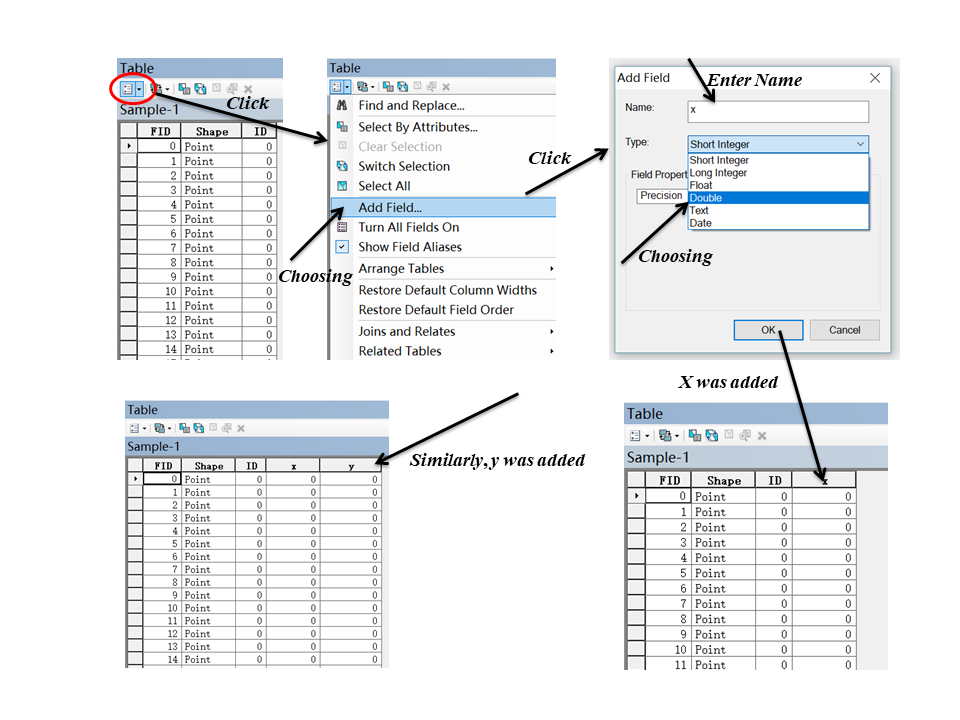


**Figure S7** Two fields were added to the attribute table in ArcMap using Arc GIS 10.3 software.


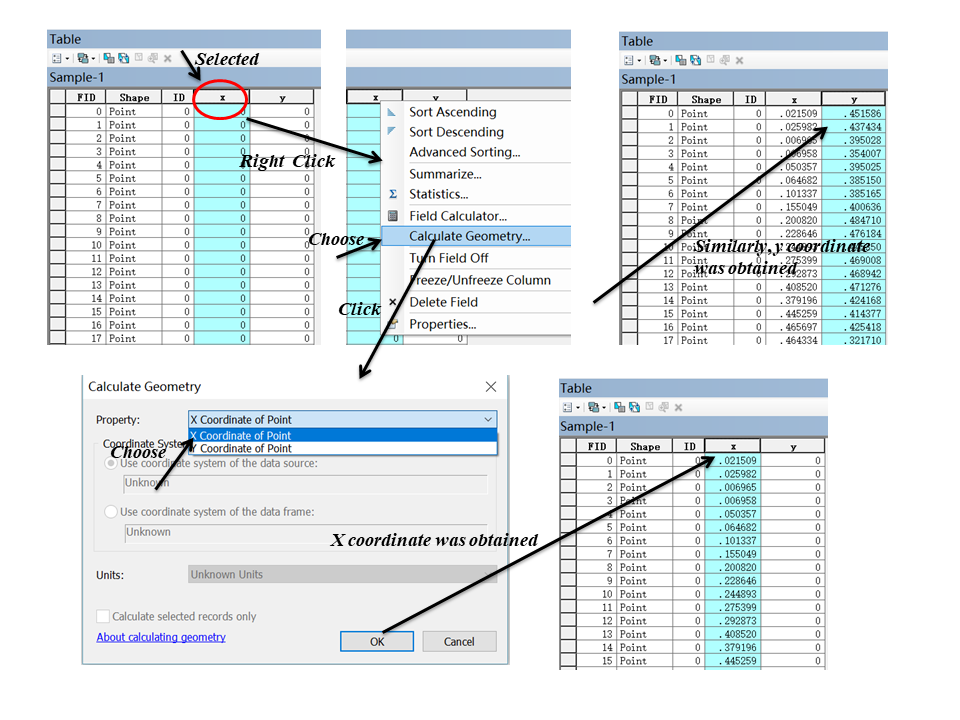


**Figure S8** The (x, y) coordinates of individuals of each species were obtained in ArcMap using Arc GIS 10.3 software.

**
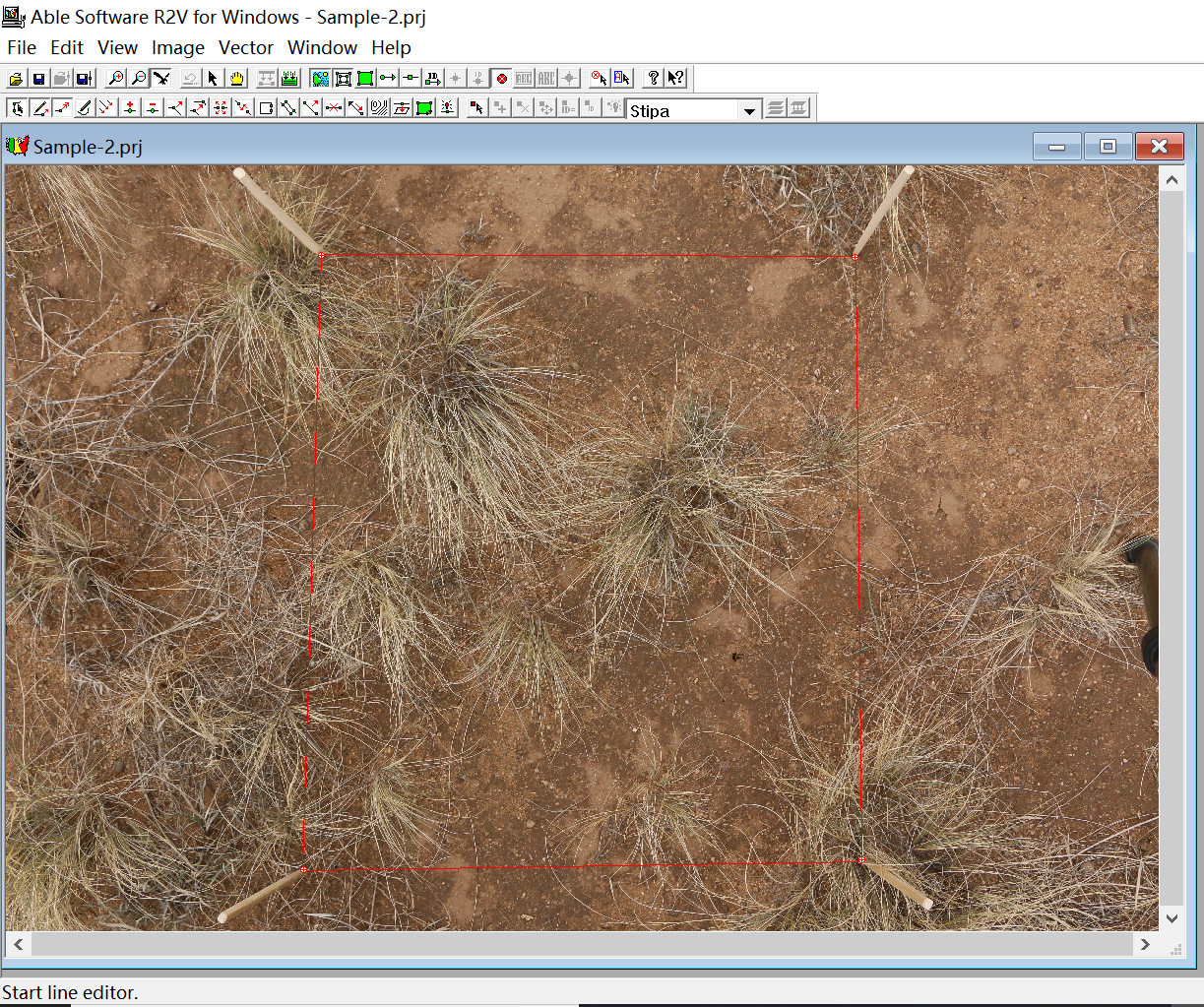
**

**Figure S9** The boundaries of sub-quadrat were added through the vertex of the sub-quadrat in R2V 7.0 software

**
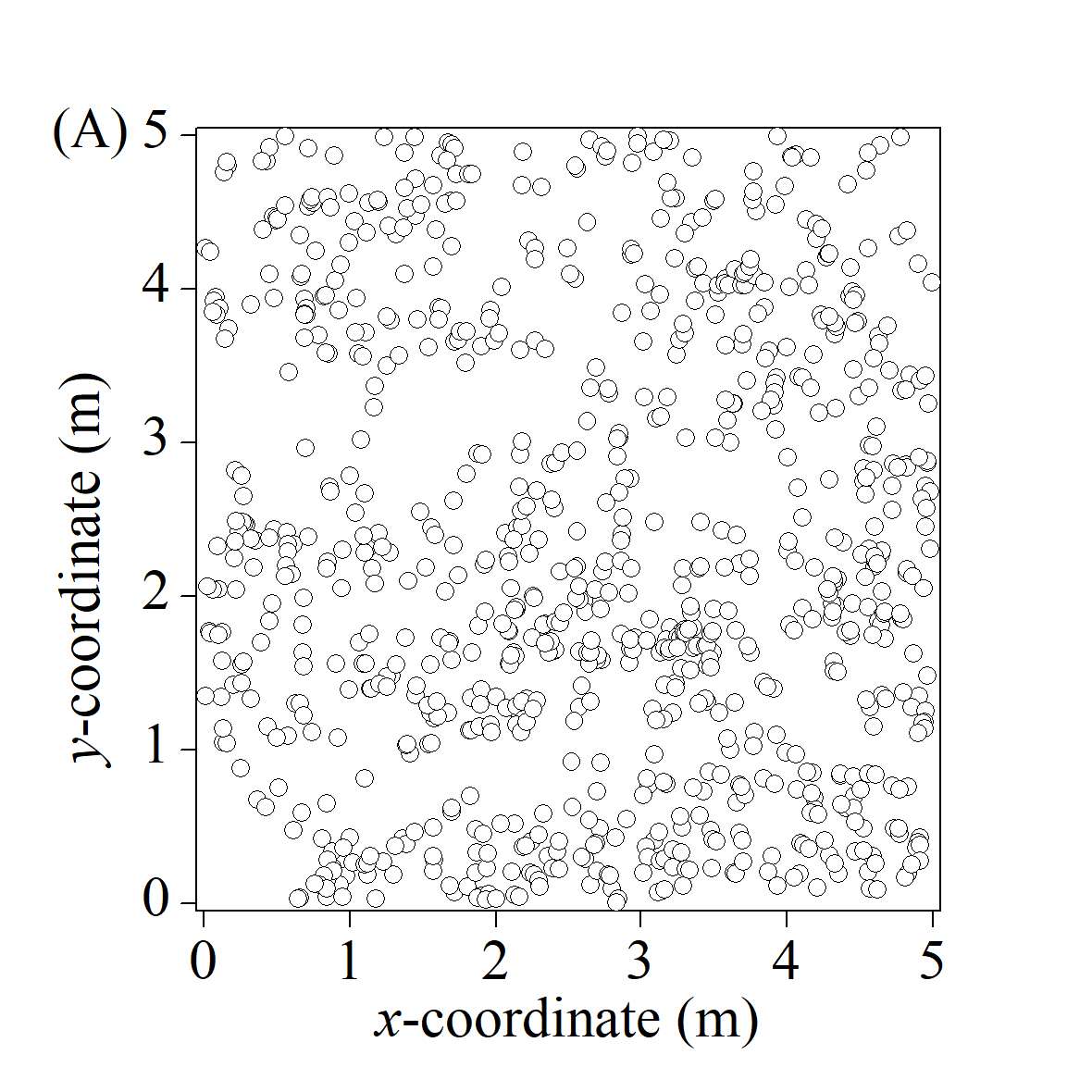

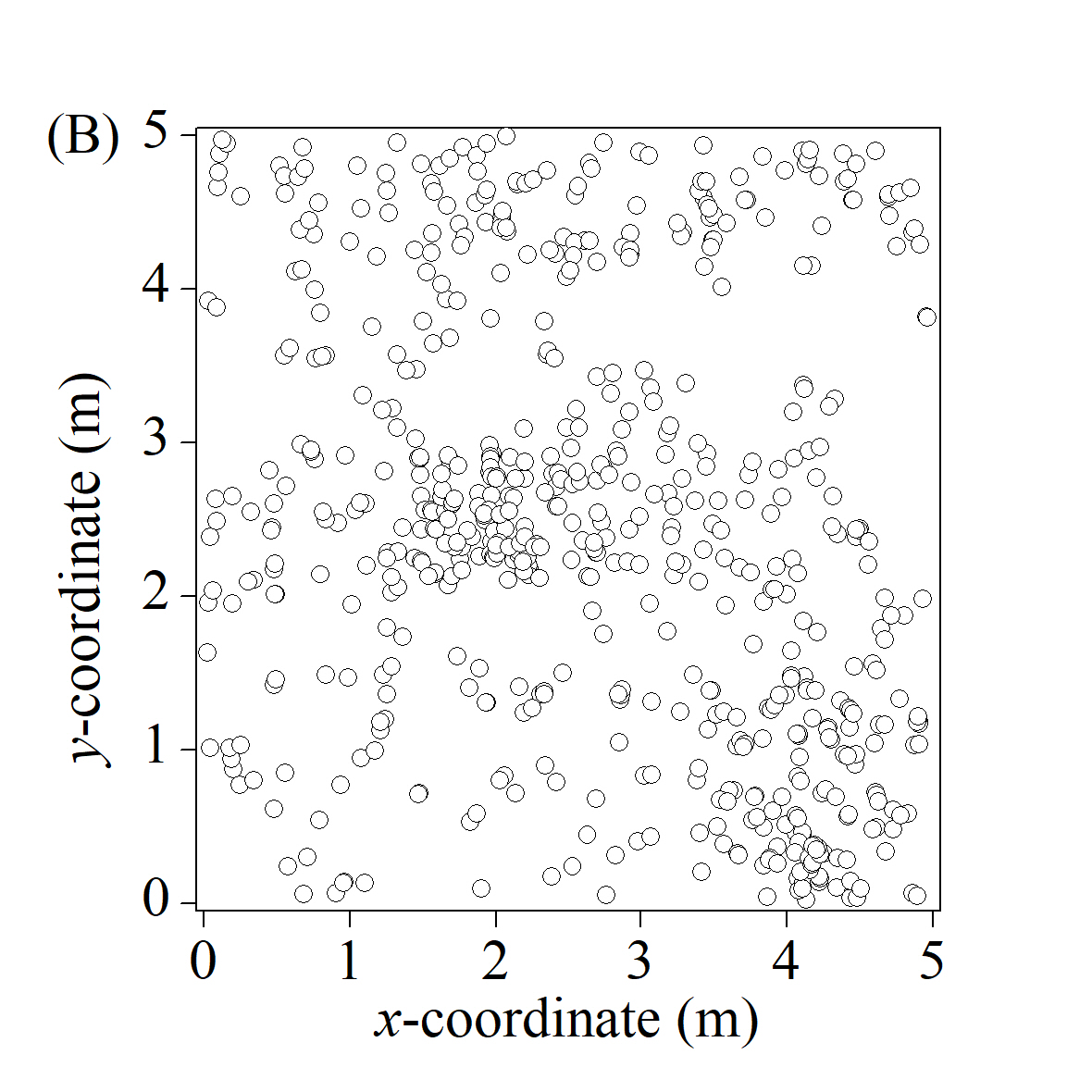
**

**
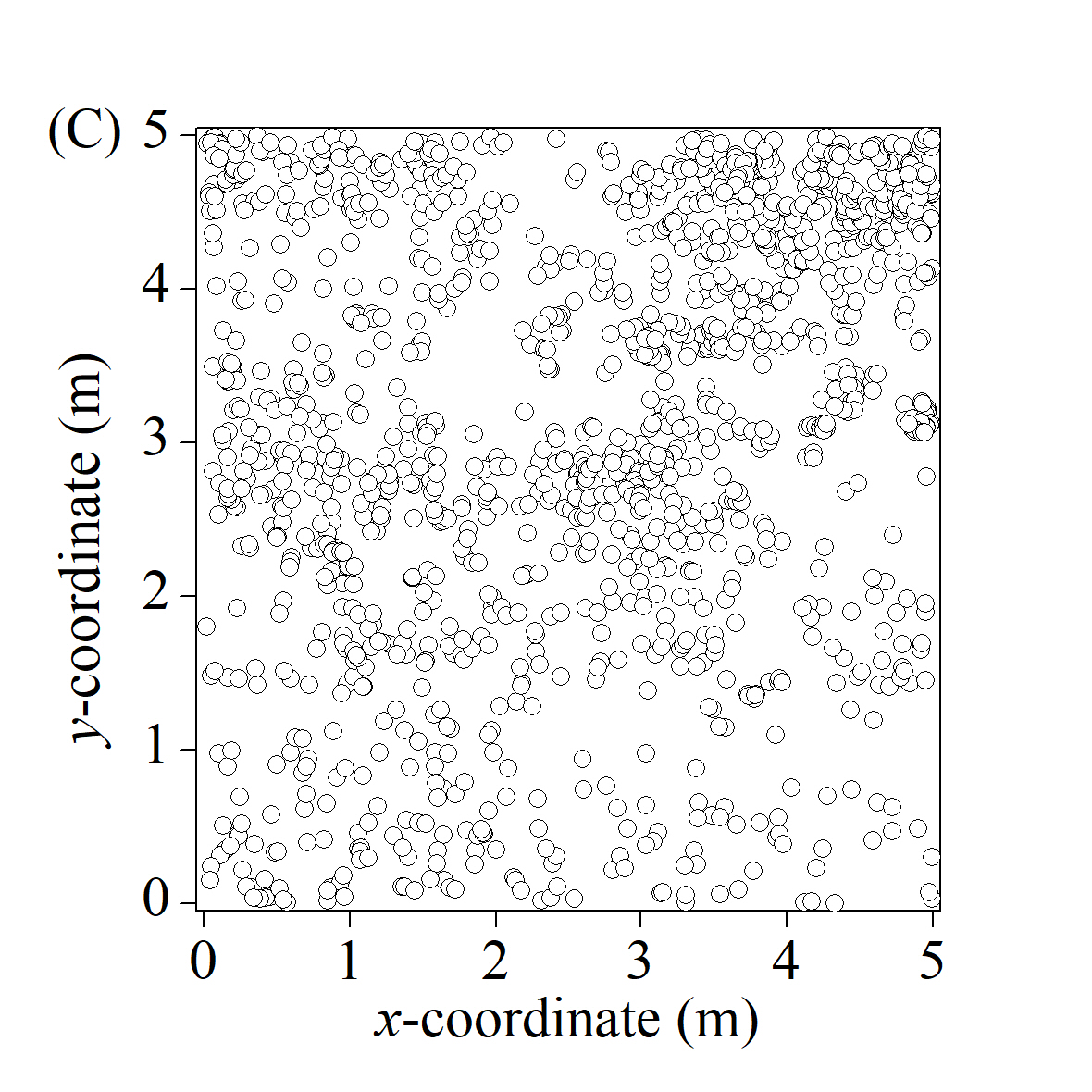

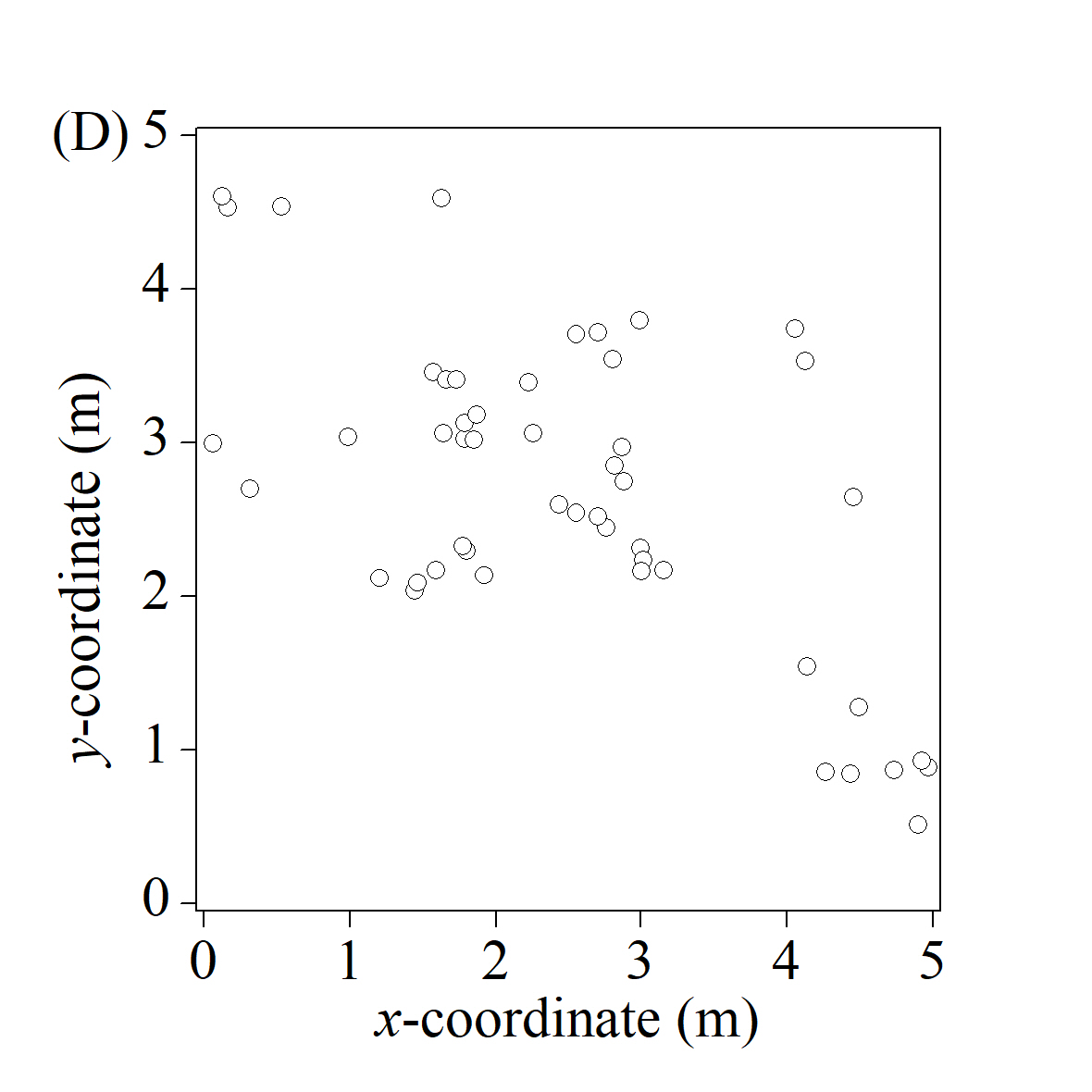
**

**
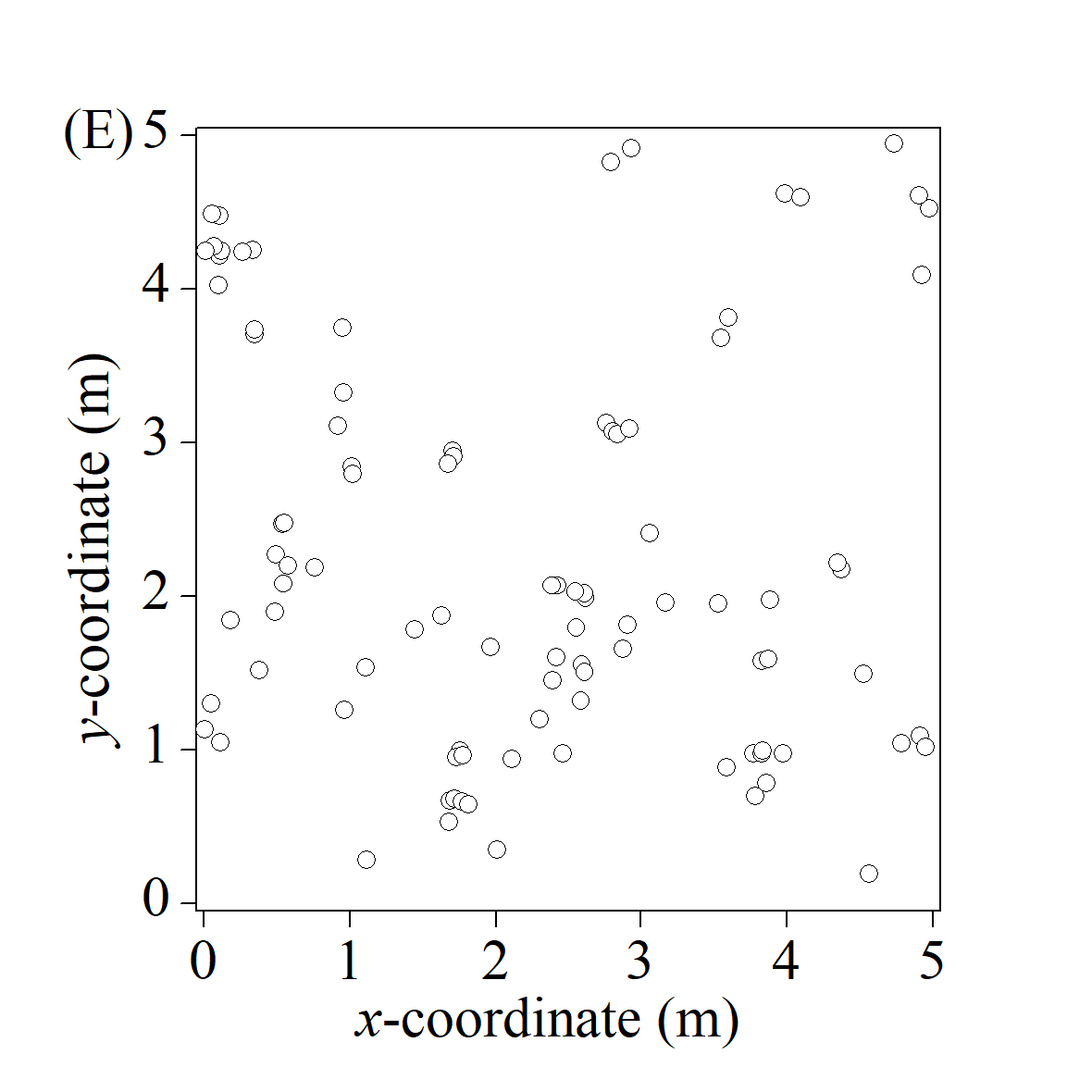

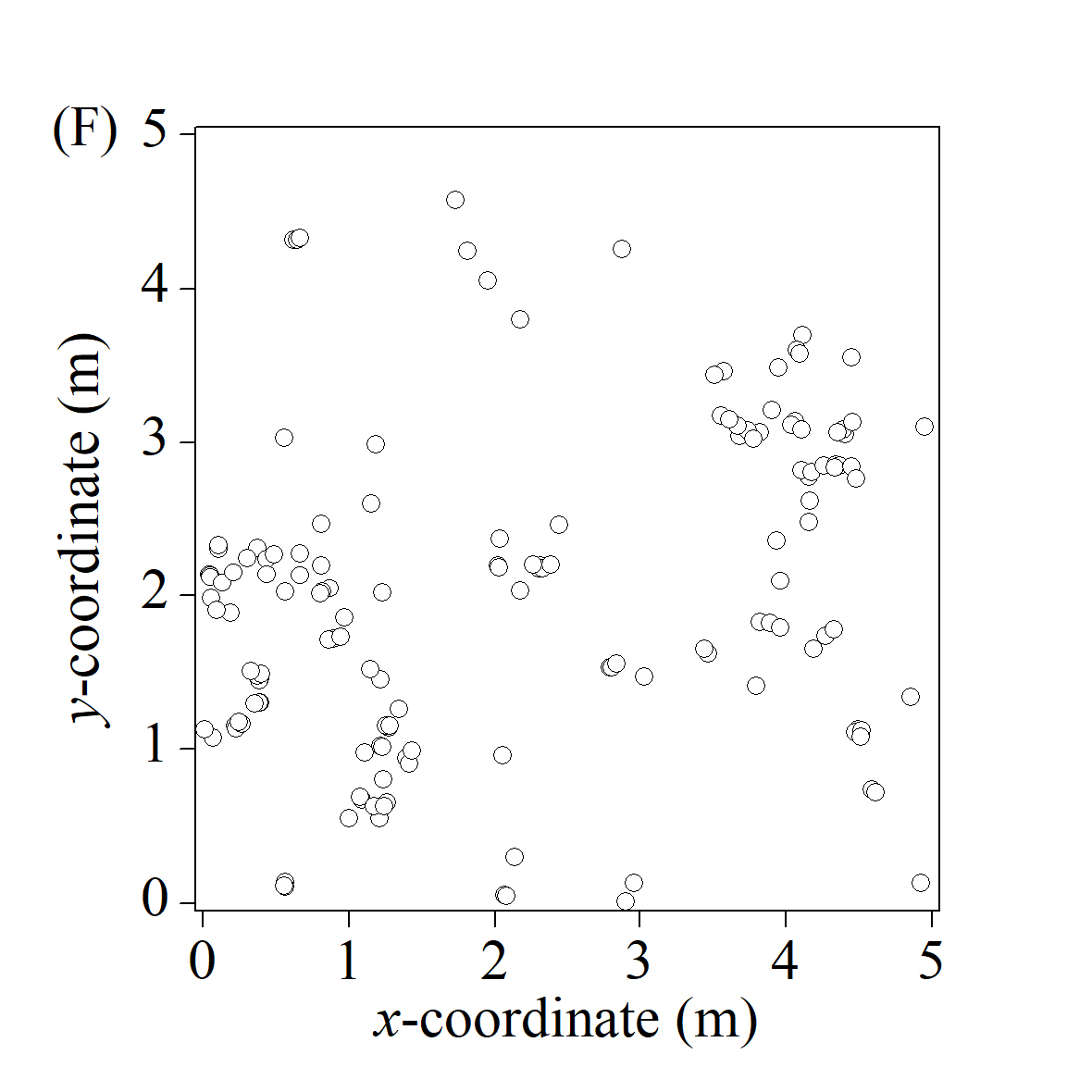
**

**Figure S10** Mapped point patterns of populations in the typical steppe by using digital photographs and geographical information system. A. *Stipa grandis*; B. *Leymus chinensis*; C. *Agropyron michnoi*; D. *Cleistogenes squarrosa*; E. *Koeleria cristata*; F. *Allium bidentatum*.

**
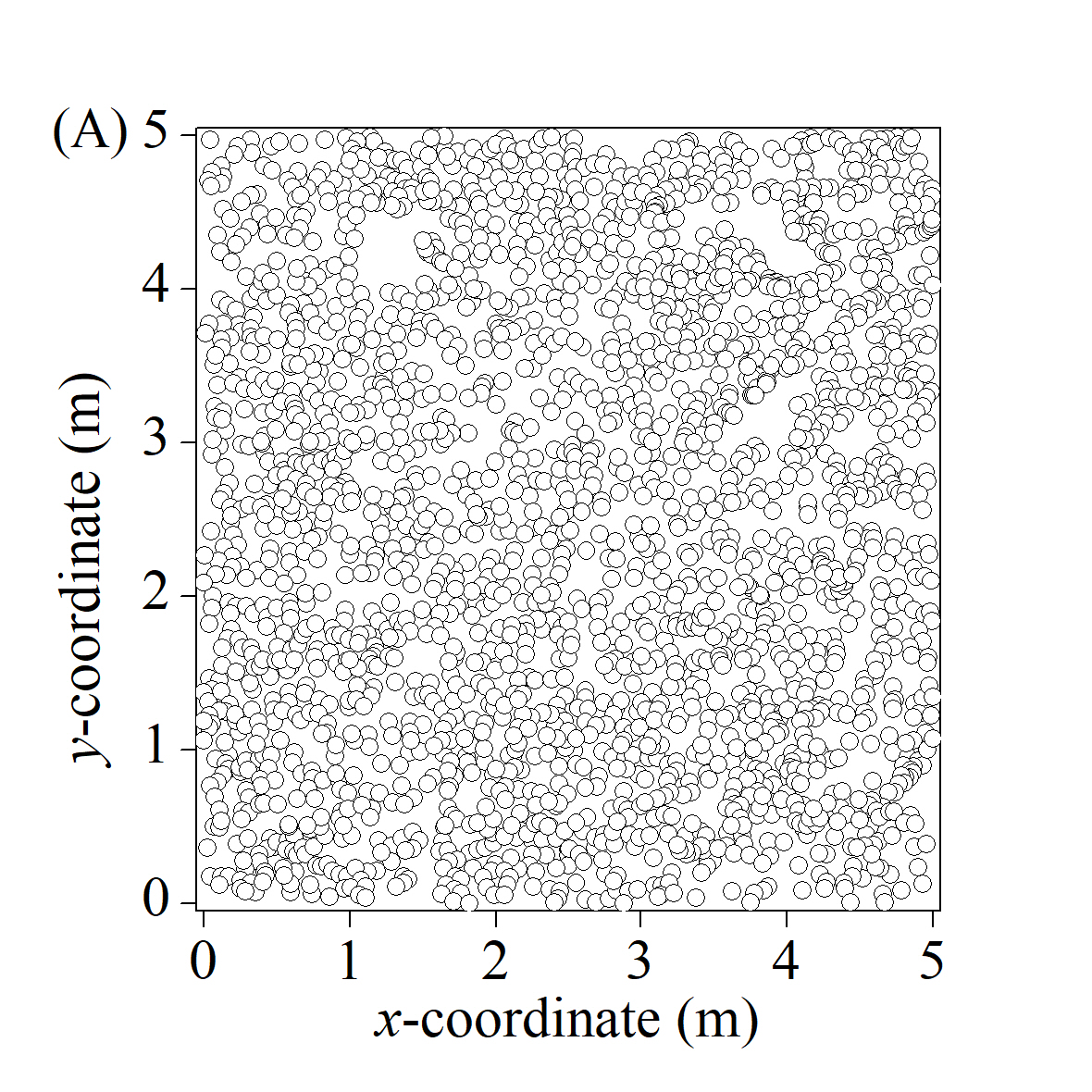

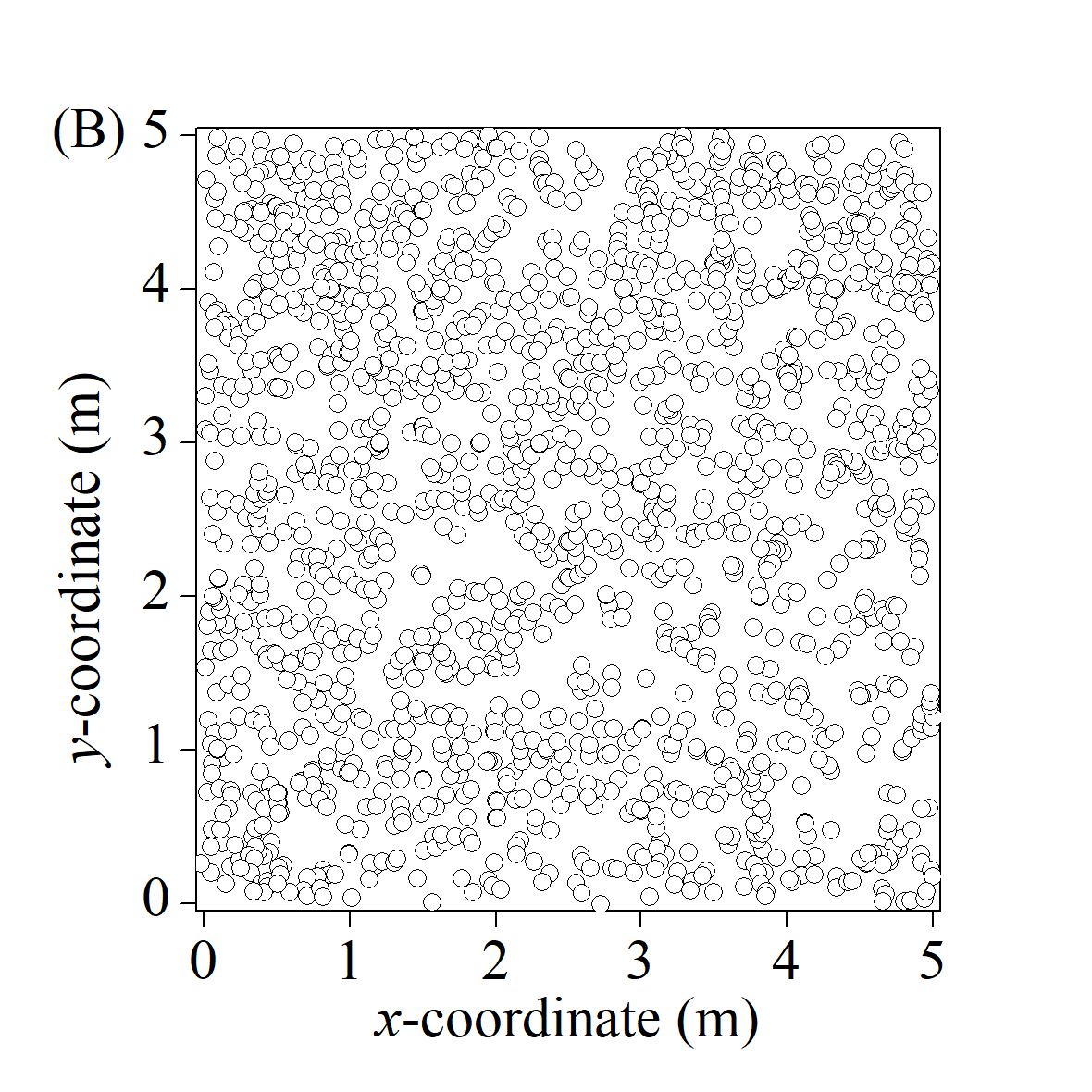
**

**Figure S11** Mapped point patterns of populations in the desert steppe by using digital photographs and geographical information system. A. *Stipa brevii*; B. *Stipa klemenzii*.
